# Prevalence of oral anticoagulant use among people with and without Alzheimer’s disease

**DOI:** 10.1186/s12877-022-03144-x

**Published:** 2022-05-28

**Authors:** Barkat Ali Babar, Mai Vu, Marjaana Koponen, Heidi Taipale, Antti Tanskanen, Raimo Kettunen, Miia Tiihonen, Sirpa Hartikainen, Anna-Maija Tolppanen

**Affiliations:** 1 School of Pharmacy, University of Eastern Finland, PO Box 1627, 70210 Kuopio, Finland; 2 Kuopio Research Center of Geriatric Care, University of Eastern Finland, Kuopio, Finland; 3 Center for Medicine Use and Safety, Faculty of Pharmacy and Pharmaceutical Sciences, Monash University, Parkville, VIC Australia; 4 Department of Clinical Neurosciences, Karolinska Institute, Stockholm, Sweden; 5 Department of Forensic Psychiatry, Niuvaniemi Hospital, University of Eastern Finland, Kuopio, Finland; 6 Public Health Solutions, National Institute for Health and Welfare, Helsinki, Finland; 7 School of Medicine, University of Eastern Finland, Kuopio, Finland

**Keywords:** Alzheimer’s disease, Oral anticoagulant, Atrial fibrillation

## Abstract

**Background:**

Although cardio- and cerebrovascular diseases are common among people with Alzheimer’s disease (AD), it is unknown how the prevalence of oral anticoagulant (OAC) use changes in relation to AD diagnosis. We investigated the prevalence of OAC use in relation to AD diagnosis in comparison to a matched cohort without AD.

**Methods:**

Register-based Medication use and Alzheimer’s disease (MEDALZ) cohort includes 70 718 Finnish people with AD diagnosed between 2005–2011. Point prevalence of OAC use (prescription register) was calculated every three months with three-month evaluation periods, from five years before to five years after clinically verified diagnosis and compared to matched cohort without AD. Longitudinal association between AD and OAC use was evaluated by generalized estimating equations (GEE).

**Results:**

OAC use was more common among people with AD until AD diagnosis, (OR 1.17; 95% CI 1.13–1.22), and less common after AD diagnosis (OR 0.87; 95% CI 0.85–0.89), compared to people without AD. At the time of AD diagnosis, prevalence was 23% and 20% among people with and without AD, respectively. OAC use among people with AD began to decline gradually two years after AD diagnosis while continuous increase was observed in the comparison cohort. Warfarin was the most common OAC, and atrial fibrillation was the most common comorbidity in OAC users.

**Conclusion:**

Decline in OAC use among people with AD after diagnosis may be attributed to high risk of falling and problems in monitoring. However, direct oral anticoagulants (DOACs) that are nowadays more commonly used require less monitoring and may also be safer for vulnerable people with AD.

**Supplementary Information:**

The online version contains supplementary material available at 10.1186/s12877-022-03144-x.

## Background

Alzheimer’s disease (AD) is a cognitive disorder that currently affects more than 26 million people globally [1]. It is the most common cause of dementia [2]. Cardiovascular risk factors and cardio- and cerebrovascular diseases have been consistently linked with a higher risk of cognitive disorders [3–5]. Atrial fibrillation is a risk factor for cognitive decline and cognitive disorders including AD [6–8], even in the absence of stroke [8]. Oral anticoagulants are a cornerstone of atrial fibrillation treatment [9, 10] and due to its high prevalence in older population [11] atrial fibrillation is one of the most common indications for oral anticoagulant use [12]. According to a systematic review, high age, increased bleeding risk, previous bleeds, falls risk, comorbidities and ability to comply with treatment influenced whether physicians would prescribe anticoagulation for atrial fibrillation [13]. Advanced age is associated with an increased risk of bleeding, which can become a reason for oral anticoagulant underutilization [14, 15], and therefore anticoagulant treatment among older people should be balanced according to its risks and benefits for users [16]. In an UK study (data from years 2000–2009) on people with incident atrial fibrillation, patients aged 80 years or more were considerably less likely to be treated with warfarin than younger patients due to higher risk scores for bleeding [17].

Currently, there is no guideline on the use of cardiovascular drugs, including oral anticoagulants, in people with dementia, and the recent guidelines of both the European Society of Cardiology (ESC) and the European Society of Hypertension (ESH) 2018 urgently call for more research on this topic [18]. ESC guidelines on management of atrial fibrillation recommend withholding oral anticoagulants in people with dementia and atrial fibrillation only in case, when compliance and adherence cannot be ensured by a caregivers [10]. According to a meta-analysis of 27 studies, people with the cognitive disorder and atrial fibrillation had 52% lower odds of using oral anticoagulants compared to people without cognitive disorder [19]. In a study on US Veteran’s Affairs database, restricted to people aged 65 and older and with atrial fibrillation, only 16% of warfarin users persisted use after dementia diagnosis, compared to 96.7% of those without dementia [20]. In an Australian population-based study people with AD were less likely to use anticoagulant than those without AD (13% vs. 18%) [21]. As these previous studies have investigated oral anticoagulant use in people who already had AD or other cognitive disorders, it is unknown whether the differences occur already before diagnosis and how they evolve over time.

To illustrate the change in prevalence of oral anticoagulant use over time, we investigated the prevalence of oral anticoagulants use among community-dwelling people with AD and their matched comparison people without AD from five years before to five years after AD diagnosis.

## Methods

### Study cohort

Medication use and Alzheimer’s disease (MEDALZ) study includes residents of Finland who received a clinically verified AD diagnosis during 2005–2011 and were community-dwelling at the time of diagnosis. The total number of people with AD was 70 718 and 65% of the study cohort were women. The average age was 80 years, ranging from 35 to 105 years [22]. To compare the prevalence of oral anticoagulant use among people with AD to those without AD, each person in AD cohort was matched with persons without AD based on age, sex and region of residence at the time of diagnosis (index date).

Social Insurance Institution (SII) of Finland maintains a Special Reimbursement Register, in which information about people who are entitled to reimbursed drugs for certain chronic conditions are recorded, AD cases were identified from this register. AD diagnosis was clinically verified and consistent with the NINCDS-ADRDA (National Institute of Neurological and Communicative Diseases and Stroke and the Alzheimer’s disease and Related Disorders Association) and DSM-IV criteria for AD (Diagnostic and Statistical Manual Fourth Edition). It includes computed tomography or magnetic resonance imaging, exclusion of alternative diagnosis and confirmation of diagnosis by geriatrician or neurologist. For medical reimbursement, people must have mild to moderate AD and if it progresses to severe stage in near future, reimbursement is not withdrawn. For people with mixed dementia, reimbursement was granted if symptoms were mainly caused by AD. MEDALZ cohort data was collected from the following nationwide registers: Prescription Register (purchased prescription drugs 1995–2015), Special Reimbursement Register (comorbidities 1972–2015), Care Registers for Health and Social Care (hospitalizations 1972–2015 and institutionalizations 1995–2015) and Statistics Finland (socioeconomic data 1972–2012 and causes of death (2005–2015) [22, 23]. The mean follow-up time for people with and without AD were 4.9 and 5.6 years, respectively [24].

### Oral anticoagulant use

Use of oral anticoagulants was identified from the Prescription register, which contains purchases of reimbursed prescription drugs for the Finnish population, by using Anatomical Therapeutic Chemical Classification System (ATC). Definition with corresponding ATC-codes of oral anticoagulants that were used, is given in supplementary table 1, defined as ATC-code B01A. Point prevalence of oral anticoagulants was evaluated every 3 months with 3-month evaluation periods from five years before and until 5 years after the index date. Persons who were dead or hospitalized/institutionalized for more than 30 days of a specific evaluation period were not included in the evaluation time period. Follow-up ended on date of death, more than 90 days hospitalization/institutionalization or five years after the index date, whichever happened first.

Drugs used in hospitals or public nursing homes are not recorded in the Prescription register. Additionally, low dose acetylsalicylic acid is not reimbursed and therefore not recorded in the register, except for combination products of acetylsalicylic acid and dipyridamole which were reimbursed. We have described use of all anticoagulants, including parenteral ones, although we do not have information whether oral and parenteral anticoagulants were used concomitantly or is the use in the same time window, due to switching from one drug substance to another. Clopidogrel, prasugrel and Direct oral anticoagulants (DOACs) had indication specific and/or periodical limitations for reimbursement (clopidogrel was reimbursed 2002–2016, prasugrel from 2010 and DOACs from 2012) and therefore, their use is limitedly recorded during those periods.

Periods of drug use were derived with the Prescription Drug Purchases to Drug Use Periods (PRE2DUP) method, which combines the drug purchases to use periods using information on personal drug purchasing patterns. This method evaluates the dose used during the period by contemplating purchased amount in Defined Daily Doses (DDD) from Prescription Register, stockpiling of drugs, purchasing regularity, and time spent in hospitals [25–27]. Persons who had used more than one oral anticoagulants in a specific period were recorded as users of all these drugs. If they had used both oral and parenteral anticoagulant, they were classified as users of both.

### Comorbidities

Data on coronary artery bypass grafting, percutaneous coronary intervention, ischemic stroke, pulmonary embolism, deep venous thrombosis, and hemorrhagic stroke, starting from one year before the index date was gathered from the Care Register for Health Care. Data on atrial fibrillation was collected from the same register from 1996 onwards. More detailed description on obtaining comorbidity data is given in supplementary table 2.

### Statistical analysis

Stata 14 (Stata Corporation, College Station, TX, USA) was used for statistical analyses. Descriptive statistics were executed by using means, standard deviation (SD) and percentages while comparison of categorical variables were done by chi-square test and t-tests for continuous variables.

Longitudinal association between AD and use of anticoagulants, before and after the index date, was evaluated by fitting population averaged panel data model with generalized estimating equations (GEE) logistic regression model. Results were adjusted for age, sex, and occupational social class. Same method was used to study the association between comorbidities over the follow-up.

## Results

### Characteristics of study population and prevalence of oral anticoagulant use on the index date

The mean age was 80 years and majority of study population were women (Table 1). At the time of AD diagnosis altogether 23% of people with AD and 20% of those without AD used oral anticoagulants, parenteral anticoagulants were seldom used. Warfarin was the most frequently used oral anticoagulant in both groups whereas, dipyridamole (alone or in combination with acetylsalicylic acid) was the most commonly used oral antiplatelet in both groups. People with AD were more likely to have been diagnosed with atrial fibrillation, ischemic stroke or deep venous thrombosis before the index date. History of coronary artery bypass grafting was more common, and percutaneous coronary intervention was less common in people with AD, although these differences were not clinically significant.

**Table 1:** Characteristics of persons with and without Alzheimer’s disease on the index date (date of Alzheimer’s disease diagnosis or corresponding matching date for comparison persons)

Total = 138 470	Alzheimer’s disease 68 609	No Alzheimer’s disease 69 861	*P*-Value
**Gender**			0.806
Women, N (%)	44 719 (65.2)	45 491(65.1)	
Men, N (%)	23 890 (34.8)	24 370 (34.9)	
**Age (y), mean ± SD**	80.0 ± 7.1	80.0 ± 7.1	0.49
**Occupational social class**			< 0.001
Managerial/professional	14 329 (20.9)	15 059 (21.6)	
Office	5 793 (8.4)	5 875 (8.4)	
Farming/forestry	13 065 (19.0)	13 604 (19.5)	
Sales/industrial/cleaning	29 203 (42.6)	27 181 (38.9)	
Unknown	6 219 (9.1)	8 142 (11.7)	
**Anticoagulant use**			< 0.001
None	52 448 (76.4)	55 685 (79.7)	
Oral	15 816 (23.1)	13 817 (19.8)	
Parenteral	200 (0.3)	258 (0.4)	
Both oral and parenteral	145 (0.2)	101 (0.1)	
**Oral anticoagulants and Oral antiplatelets**			
Warfarin	10 777 (15.7)	9 471 (13.6)	< 0.001
Dipyridamole^a^	4 422 (6.4)	3 486 (5.0)	< 0.001
Clopidogrel	1 055 (1.5)	1 185 (1.7)	0.019
Prasugrel	-^b^	0	N.A^c^
**Direct oral anticoagulants**			
Dabigatran	16 (0.02)	22 (0.03)	0.359
Rivaroxaban	15 (0.02)	28 (0.04)	0.054
**Comorbidity**			
Atrial fibrillation	10 211 (14.9)	8 550 (12.2)	< 0.001
Coronary artery bypass grafting	2 370 (3.5)	2 247 (3.2)	0.014
Percutaneous coronary intervention	1 626 (2.4)	1 831 (2.6)	0.003
Ischemic stroke	4 546 (6.6)	3 921 (5.6)	< 0.001
Pulmonary embolism	925 (1.3)	948 (1.4)	0.88
Deep venous thrombosis	1 310 (1.9)	1 175 (1.7)	0.001
Hemorrhagic stroke	164 (0.2)	135 (0.2)	0.066

^a^Dipyridamole alone or in combination with acetylsalicylic acid

^b^Number of individuals < 5, exact number not reported to comply with restrictions regarding data reporting

^c^*N. A* Not applicable

**Fig. 1:**
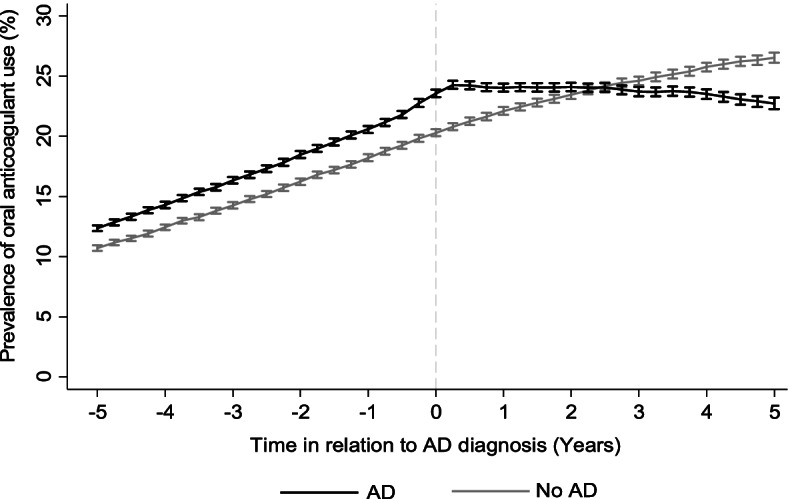
Prevalence of oral anticoagulant use among people with and without Alzheimer’s disease (AD) in relation to AD diagnosis

Before the index date, the use of oral anticoagulants was more common among people with AD (unadjusted OR = 1.17; 95% CI 1.13–1.22) compared to people without AD but the difference stabilized at the time of AD diagnosis (Fig. 1). Among people with AD, the prevalence of oral anticoagulant use began to decrease slowly two years after the AD diagnosis, while the prevalence continued to increase among people without AD throughout the entire study period. Consequently, the oral anticoagulant use was less common among people with AD after the index date (unadjusted OR = 0.87; 95% CI 0.85–0.89).

Characteristics of warfarin users and non-users are shown in Table 2. Atrial fibrillation, ischemic stroke, coronary artery bypass grafting, and percutaneous coronary intervention were more common in warfarin users than non-users of warfarin.

**Table 2:** Characteristics of users and non-users of warfarin on the index date. Comorbidities were defined in relation to the index date

**Total: 138 470**	**Warfarin Users (20 248) N (%)**		*P*-Value	**Non- users of warfarin (118 222) N (%)**		*P*-Value
	People with AD 10 777 (53.2)	People without AD 9 471 (46.8)		People with AD 57 832 (48.9)	People without AD 60 390 (51.1)	
**Gender**			0.007			0.057
Women, n (%)	6 438 (59.7)	5 834 (61.6)		38 281 (66.2)	39 657 (65.7)	
Men, n (%)	4 339 (40.3)	3 637 (38.4)		19 551 (33.8)	20 733 (34.3)	
**Age (y), mean ± SD**	81.8 ± 5.6	82.1 ± 5.6	0.0015	79.7 ± 7.3	79.7 ± 7.2	0.72
**Occupational social class**			0.011			< 0.001
Managerial/Professional	2 051 (19.0)	1 793 (18.9)		12 278 (21.2)	13 266 (22.0)	
Office	742 (6.9)	692 (7.3)		5 051 (8.7)	5 183 (8.6)	
Farming/Forestry	2 372 (22.0)	2 186 (23.1)		10 693 (18.5)	11 418 (18.9)	
Sales/Industrial/Cleaning	4 686 (43.5)	3 911 (41.3)		24 517 (42.4)	23 270 (38.5)	
Unknown	926 (8.6)	889 (9.4)		5 293 (9.2)	7 253 (12.0)	
**Comorbidity**						
**Atrial fibrillation**			< 0.001			< 0.001
< 6 months before	2 272 (21.1)	1 376 (14.5)		990 (1.7)	544 (0.9)	
6–12 months before	684 (6.3)	620 (6.5)		318 (0.5)	274 (0.5)	
More than 1 year before	3 895 (36.1)	3 856 (40.7)		2 052 (3.5)	1 880 (3.1)	
**Coronary artery bypass grafting**			0.001			0.003
< 6 months before	19 (0.2)	45 (0.5)		27 (0.05)	55 (0.1)	
6–12 months before	19 (0.2)	16 (0.2)		31 (0.1)	49 (0.1)	
**Percutaneous coronary intervention**			0.056			0.001
< 6 months before	28 (0.3)	34 (0.4)		141 (0.2)	205 (0.3)	
6–12 months before	19 (0.2)	30 (0.3)		86 (0.2)	119 (0.2)	
**Ischemic stroke**			0.050			< 0.001
< 6 months before	278 (2.6)	196 (2.1)		632 (1.1)	403 (0.7)	
6–12 months before	133 (1.2)	110 (1.2)		248 (0.4)	203 (0.3)	
**Pulmonary embolism**			0.903			0.018
< 6 months before	111 (1.0)	99 (1.1)		50 (0.1)	31 (0.1)	
6–12 months before	50 (0.5)	48 (0.5)		23 (0.04)	37 (0.1)	
**Deep venous thrombosis**			0.094			0.172
< 6 months before	104 (1.0)	65 (0.7)		52 (0.1)	37 (0.1)	
6–12 months before	30 (0.3)	27 (0.3)		35 (0.1)	32 (0.1)	
**Hemorrhagic stroke**			N.A^a^			0.146
< 6 months before	-^b^	-^b^		17 (0.03)	10 (0.02)	
6–12 months before	-^b^	-^b^		-^b^	9 (0.01)	

^a^*N. A* Not applicable

^b^Number of individuals < 5, exact number not reported to comply with restrictions regarding data reporting

### Characteristics of oral anticoagulant users

Characteristics of oral anticoagulant users and non-users on the index date are described in Table 3. Atrial fibrillation was the most common comorbidity among oral anticoagulant users; overall prevalence on index date was 45% among oral anticoagulant users with AD and 44% among oral anticoagulant users without AD (Table 3). In the longitudinal analyses (Table 4), the strongest association were observed with recent (recorded < 6 months before the beginning of specific time window) percutaneous coronary intervention, pulmonary embolism, and deep venous thrombosis among both people with and without AD.

**Table 3:** Characteristics of users and non-users of oral anticoagulant on index date. Comorbidities were defined in relation to the index date

Total = 138 470	People with Alzheimer’s Disease (68 609) N (%)			People without Alzheimer’s Disease (69 861) N (%)		
	Users *N* = 15 961	Non-Users *N* = 52 648	*P*- Value	Users *N* = 13 918	Non-users *N* = 55 943	*P*- Value
**Gender**			< 0.001			< 0.001
Women, n (%)	9 521 (59.7)	35 198 (66.9)		8 582 (61.7)	36 909 (66.0)	
Men, n (%)	6 440 (40.3)	17 450 (33.1)		5 336 (38.3)	19 034 (34.0)	
**Age (y), mean ± SD**	81.6 ± 5.8	79.5 ± 7.4	< 0.001	82.0 ± 5.7	79.5 ± 7.3	< 0.001
**Occupational social class**			< 0.001			< 0.001
Managerial/Professional	3 082 (19.3)	11 247 (21.4)		2 662 (19.1)	12 397 (22.2)	
Office	1 123 (7.0)	4 670 (8.9)		1 037 (7.5)	4 838 (8.6)	
Farming, Forestry	3 435 (21.5)	9 630 (18.3)		3 209 (23.1)	10 395 (18.6)	
Sales/Industrial/Cleaning	6 940 (43.5)	22 263 (42.3)		5 677 (40.8)	21 504 (38.4)	
Unknown	1 381 (8.7)	4 838 (9.2)		1 333 (9.6)	6 809 (12.2)	
**Comorbidity**						
**Atrial fibrillation**			< 0.001			< 0.001
< 6 months before	2 384 (14.9)	878 (1.7)		1 454 (10.4)	466 (0.8)	
6–12 months before	720 (4.5)	282 (0.5)		645 (4.6)	249 (0.4)	
More than 1 year before	4 107 (25.7)	1 840 (3.5)		4 033 (29.0)	1 703 (3.0)	
**Coronary artery bypass grafting**			< 0.001			< 0.001
< 6 months before	24 (0.2)	22 (0.04)		59 (0.4)	41 (0.07)	
6–12 months before	23 (0.1)	27 (0.05)		23 (0.2)	42 (0.08)	
**Percutaneous coronary intervention**			< 0.001			< 0.001
< 6 months before	145 (0.9)	24 (0.05)		207 (1.5)	32 (0.06)	
6–12 months before	64 (0.4)	41 (0.08)		92 (0.7)	57 (0.1)	
**Ischemic stroke**			< 0.001			< 0.001
< 6 months before	610 (3.8)	300 (0.6)		423 (3.0)	176 (0.3)	
6–12 months before	270 (1.7)	111 (0.2)		231 (1.7)	82 (0.1)	
**Pulmonary embolism**			< 0.001			< 0.001
< 6 months before	119 (0.8)	42 (0.08)		106 (0.8)	24 (0.04)	
6–12 months before	50 (0.3)	23 (0.04)		53 (0.4)	32 (0.06)	
**Deep venous thrombosis**			< 0.001			< 0.001
< 6 months before	108 (0.7)	48 (0.09)		69 (0.5)	33 (0.06)	
6–12 months before	33 (0.2)	32 (0.06)		28 (0.2)	31 (0.06)	
**Hemorrhagic stroke**			0.39			0.99
< 6 months before	6 (0.04)	15 (0.03)		-^a^	9 (0.02)	
6–12 months before	-^a^	-^a^		-^a^	8 (0.01)	

^a^Number of individuals < 5, exact number not reported to comply with restrictions regarding data reporting

**Table 4:** Longitudinal association between comorbidities and oral anticoagulant use (OR 95% CI) during the entire follow-up. Data on comorbidities were defined time-dependently for the entire follow-up

Comorbidity (Reference: no disease)	People with AD OR (95% CI)	People without AD OR (95% CI)
**Atrial fibrillation**		
< 6 months before	5.12 (4.90–5.36)	7.22 (6.87–7.57)
6–12 months before	4.65 (4.42–4.88)	6.55 (6.22–6.90)
More than one year before	4.34 (4.13–4.56)	5.79 (5.49–6.10)
**Coronary artery bypass grafting**		
< 6 months before	2.68 (2.40–3.00)	3.05 (2.79–3.35)
6–12 months before	1.36 (1.21–1.53)	1.48 (1.34–1.63)
**Percutaneous coronary intervention**		
< 6 months before	13.58 (12.10–15.25)	17.99 (16.35–19.79)
6–12 months before	4.51 (4.07–4.99)	5.54 (5.10–6.01)
**Ischemic stroke**		
< 6 months before	2.40 (2.30–2.51)	2.95 (2.81–3.10)
6–12 months before	2.39 (2.29–2.51)	2.79 (2.65–2.93)
**Pulmonary embolism**		
< 6 months before	6.59 (5.93–7.33)	6.82 (6.13–7.58)
6–12 months before	3.70 (3.35–4.10)	3.52 (3.18–3.89)
**Deep venous thrombosis**		
< 6 months before	7.54 (6.81–8.35)	5.86 (5.23–6.57)
6–12 months before	2.81 (2.57–3.08)	2.35 (2.11–2.63)
**Hemorrhagic stroke**		
< 6 months before	1.20 (0.97–1.49)	1.45 (1.11–1.90)
6–12 months before	0.95 (0.71–1.26)	1.14 (0.84–1.54)

Men and older people were more likely to use oral anticoagulants among people with and without AD in both cross-sectional and longitudinal analyses. In longitudinal analyses, the association of age and sex were similar before and after AD diagnosis (OR, 95% CI for oral anticoagulant use per one year increase in age was 1.05, 1.05–1.06 before AD diagnosis and 1.05, 1.04–1.05 after AD diagnosis, OR, 95% CI for oral anticoagulant use in men compared to women 1.36, 1.31–1.41 before AD diagnosis 1.35, 1.32–1.39 after AD diagnosis).

## Discussion

Previous studies have demonstrated that people with cognitive disorders are less likely to use oral anticoagulants [19, 21] and discontinuation often occurs at dementia diagnosis [20]. However, as development of cognitive disorder is a long process, so investigation of changes in drug utilization prior to diagnosis is relevant. Our study extends the previous knowledge by reporting the changes in the prevalence of oral anticoagulant use among people with and without AD in relation to AD diagnosis. Before AD diagnosis, prevalence of oral anticoagulant use was higher among people with AD compared to people without AD. However, among people with AD prevalence increased until the time of AD diagnosis, stabilized around the time of diagnosis and then started to decrease gradually after two years of diagnosis. On the contrast, use of oral anticoagulants continued to increase among people without AD over the whole follow-up.

Nearly one out of four persons with AD used oral anticoagulants at the time of AD diagnosis and warfarin was the most commonly used oral anticoagulant in our study. This prevalence is higher than that observed in an Australian study of people with dispensed anti-dementia medication. In that study, the prevalence of warfarin use among anti-dementia drug users was 8% in 2013/2014 and 12% in 2016/2017 [28]. On the other hand, a study based on Spanish dementia registry reported higher prevalence (49%) of anticoagulant use compared to our study [29]. The difference might be due to wider definition of oral anticoagulant use in the Spanish study (entire ATC category B01, which includes also parenteral anticoagulants and acetylsalicylic acid). Furthermore, Spanish study included all types of dementia, although people with AD were in majority (58%). The between-country differences in prevalence of anticoagulant use can also be explained by different health care and reimbursement systems in each country.

Prevalence of warfarin use in our study was lower compared to findings from Swedish dementia registry (16% vs. 21.6%). This might be explained by difference in the population as in Swedish study included only persons with atrial fibrillation [30]. At the time of index dates (years 2005–2011), DOACs were just entering to market, had strictly limited reimbursement status and thus were expensive for the patients which may also explain their lower prevalence compared with warfarin in our study. Nowadays, a shift from warfarin to DOACs has occurred [31], but this was not yet visible during the follow-up of this study. Therefore, a less steep decline after AD diagnosis may be observed with more recent data, because DOACs are recommended for atrial fibrillation in old persons irrespective of age, risk of falling, and cognitive impairment, in line with this, a recent review reported that availability of DOACs has increased the proportion of older individuals using oral antithrombotics [32].

The higher prevalence of oral anticoagulant use before AD diagnosis in our study likely reflects the burden of atrial fibrillation, other cardiovascular diseases, and cardiovascular risk factors among people with AD. The decline in oral anticoagulant use after AD diagnosis may be partly explained by difficulties in monitoring and managing anticoagulant treatment in older people, as bleeding risk increases abruptly with advancing age and AD [33–35]. People with AD and other dementias have also a higher risk for adverse effects and events like falls [19, 36] and are more likely to be frail, which may have affected the decision to deprescribe oral anticoagulants although the recent consensus paper STOPPFrail (Screening Tool of Older Persons Prescriptions in Frail adults with limited life expectancy) did not reach consensus on anticoagulant deprescription in people with limited life expectancy [37]. It should be noted that we were not able to assess whether discontinuation was due to deprescribing, and we also had no information on frailty. Because AD was diagnosed in mild or moderate stage, over two thirds of the AD cohort survived over four years [24]. Still, frailty and limited life expectancy of the AD cohort may also explain the results.

Bleeding and dementia are among the main reasons for not prescribing oral anticoagulants for atrial fibrillation patients [31]. However, according to ESC, anticoagulants should also be used among people with dementia and atrial fibrillation unless compliance and adherence cannot be ensured by a caregiver [10]. In the Finnish health care system, administration and monitoring of medication use can be managed also by homecare services (nurses) [38]. Our finding on the lower prevalence of oral anticoagulant use among people with AD is in accordance with a systemic review and meta-analysis which found lower odds of using oral anticoagulants for people with dementia than those without dementia [19].

The strength of our study is nationwide cohort of people with clinically verified AD diagnosis and the opportunity to investigate temporal changes in prevalence of oral anticoagulant use in relation to AD diagnosis. Data on oral anticoagulant use were obtained from the Prescription register omitting recall and selection biases. Further, we utilized dispensing data which reflects usage better than prescription data. One limitation of this study is that we had no data on oral anticoagulant use in hospitals or nursing homes, that’s why individuals who stayed in these facilities were censored. Therefore, our findings are not generalizable to institutionalized people. We also have no data on the low dose acetylsalicylic acid because it is not reimbursed. Additionally, other anticoagulants than warfarin and dipyridamole have had lacked or limited reimbursement for certain indications and /or for limited time, therefore use of those anticoagulants might be underestimated. Further, information related to factors which may influence the oral anticoagulant use e.g., severity of comorbidities, frailty status, support from family members for oral anticoagulant use, were not available in our data.

## Conclusion

The prevalence of oral anticoagulant use among people with AD began to decline steadily two years after AD diagnosis, while continued to increase among people without AD. This decrease may be attributed to the risks such as increased risk of falling, as well as problems in drug monitoring among people with AD. DOACs that are nowadays more commonly used require less monitoring and may also be safer for vulnerable people with AD so the decrease may be less steep with more recent data.

## Supplementary Information

**Additional file 1:** **Supplementary Table 1.** Anatomical therapeutic Chemical (ATC) classification of oral anticoagulants used in this study. **Supplementary Table 2.** Definitions and data sources of comorbidities.

## Data Availability

The data that support the findings of this study are available from the corresponding author, but restrictions apply to availability of these data, so they are not publicly available. Data are however available from AMT (anna-maija.tolppanen@uef.fi) upon reasonable request and with permission of the register maintainers.

## Abbreviations

AD: Alzheimer’s disease
OAC: Oral anticoagulant
MEDALZ: Medication use and Alzheimer’s disease
ESC: European society of cardiology
ESH: European society of hypertension
SII: Social insurance institution
NINCDS-ADRDA: National Institute of Neurological and Communicative Diseases and Stroke and the Alzheimer’s disease and Related Disorders Association
ATC: Anatomical therapeutical chemical classification system
DOACs: Direct oral anticoagulants
PRE2DUP: Prescription drug purchases to drug use periods
DDD: Defined daily doses
SD: Standard deviation
GEE: Generalized estimating equations
