# Supplementary material for: Prevalence of oral anticoagulant use among people with and without Alzheimer’s disease
Source: BMC Geriatr. 2022 May 28;22:464. doi: 10.1186/s12877-022-03144-x (PMC9148467; doi:10.1186/s12877-022-03144-x)
Supplement: Supplementary file 1 — Additional file 1: Supplementary Table 1. Anatomical therapeutic Chemical (ATC) classification of oral anticoagulants used in this study. Supplementary Table 2. Definitions and data sources of comorbidities. [file 12877_2022_3144_MOESM1_ESM.docx]

**Supplementary Table 1**: Anatomical therapeutic Chemical (ATC) classification of oral anticoagulants used in this study.

| **ATC Code** | **Drug Name** |
| --- | --- |
| **Oral anticoagulants and oral antiplatelets** | |
| B01AA03 | Warfarin |
| B01AC30 | Combinations^*^ |
| B01AC07 | Dipyridamole |
| B01AC04 | Clopidogrel |
| B01AC22 | Prasugrel |
| **Direct oral anticoagulants** | |
| B01AE07 | Dabigatran |
| B01AF01 | Rivaroxaban |
| B01AE05 | Ximelagatran |
| **Parenteral anticoagulants** | |
| B01AB05 | Enoxaparin |
| B01AB04 | Dalteparin |
| B01AX05 | Fondaparinux |
| B01AB10 | Tinzaparin |

^*^Acetylsalicylic acid in combinations with dipyridamole

.

**Supplementary Table 2**: Definitions and data sources of comorbidities.

| **Comorbidity** | **Data sources and coding** | **Years** |
| --- | --- | --- |
| Atrial fibrillation | Care register for health care  ICD-10 I48 | 1996-2015 |
| Coronary artery bypass grafting | Care register for health care NOMESCO^*^ FNA, FNC, FNE, code AA in the extra sheet of cardiac patient | 1996-2015 |
| Percutaneous coronary intervention | Care register for health care NOMESCO FNG00, FNG10, FN1AT, FN1BT, FN1YT, codes AN2, AN3, AN4 in the extra sheet of cardiac patient | 1996-2015 |
| Ischemic stroke | Care register for health care  ICD-10 I63 | 1996-2015 |
| Pulmonary embolism | Care register for health care  ICD-10 I26 | 1996-2015 |
| Deep venous thrombosis | Care register for health care  ICD-10 I80 | 1996-2015 |
| Hemorrhagic stroke | Care register for health care  ICD-10 I60, I61, I62 | 1996-2015 |

*Nordic Medico-Statistical Committee
